# Scoliosis: Brace treatment – from the past 50 years to the future

**DOI:** 10.1097/MD.0000000000030556

**Published:** 2022-09-16

**Authors:** F. Landauer, Klemens Trieb

**Affiliations:** a Department of Orthopaedic and Trauma Surgery, Paracelsus Medical University Salzburg, Salzburg, Austria; b Computed Tomography Research Group, University of Applied Sciences Upper Austria, Wels, Austria.

**Keywords:** brace treatment, future, past, scoliosis

## Abstract

This paper deals with scoliosis treatment over the past 50 years. The review of the literature from the point of view of the current formation of opinion. From conservative forms of treatment, the pendulum has swung to surgical measures. To visualize this temporarily rejection of conservative treatment is the goal of this article.

**Materials and Methods:**

A review of the literature over the last 50 years was performed from the perspective of current opinion, this with a pinch of personal experience in bracing and scoliosis surgery since 1972. The MESH terms (scoliosis, idiopathic scoliosis, adolescent idiopathic scoliosis) are presented in their number in a flow diagram and the publications on conservative therapies (brace, physiotherapy) are compared to surgical therapies (surgery).

Opinions of “eminences” in the 1980s have been replaced by the rules of evidence-based medicine (EBM) at end of the 1990s. This transition will be visualized in the graph of PubMed statistics. In a statement, the future scoliosis treatment is derived from history.

**Results:**

The total number of publications shows a ratio of brace to surgery of 13.9% and physiotherapy to surgery of 6.7% for the MESH terms “scoliosis”. When “scoliosis” is supplemented with “idiopathic”, the brace to surgery ratio changes from 24.5% and physiotherapy to surgery 8.2%. Focusing on adolescent scoliosis the addition of “adolescent” changes the brace to surgery ratio from 24.8% and physiotherapy to surgery 8.1%. In the total number of publications, “adolescent idiopathic scoliosis” is treated by 25.26%. The patient numbers of our own scoliosis outpatient clinic (1482 patients) over the last 15 years show a ratio of brace (Cobb angle 20°–50° brace-indication) to surgery (Cobb angle >50° indication to surgery) of 1 to 0.06. The scientific focus on surgical therapy is evident from the figures of PubMed mentioned. The number of conservative publications shows a depression in the 1990s. In the remainder of this article, opinion-forming developments are outlined and supported by literature citations, responsible for the recovery of publications on conservative scoliosis treatment. New technologies provide additional treatment options.

**Conclusions:**

In this sense, brace therapy is a success story with a future in the digital world of AI (artificial intelligence), mathematical model calculations, and production perhaps from the 3D printer. The central message from the history of the last 50 years is: “The scientific review of treatment results is essential for the further acceptance of brace treatment.”

## 1. Introduction

In scoliosis treatment, a differentiated diagnosis of the cause of scoliosis is paramount. The term “idiopathic” scoliosis is used too generously and clouds the view of the expected chances of success in brace treatment. Adolescent idiopathic scoliosis is a multifactorial disease with intrinsic and extrinsic alterations. Not every scoliosis is amenable to brace treatment. Accepting this in the absence of alternatives is often difficult. The first correction result in the brace provides the decisive answer for predicting the long-term result. Only if these questions can be answered positively does patient compliance become the decisive factor.

In many cases, the cause of scoliosis is considered unknown and therefore idiopathic. This poorly differentiated view leads to a wide range of different progression courses with treatment outcomes that are difficult to predict. In contrast, a simple differentiation into intraspinal and extraspinal causes already leads to a decisive improvement for the prediction of the course of treatment.

A finite element model of the spinal column including growth dynamics suggests that accelerated growth profiles may encourage supplementary scoliotic progression and, thus, may pose as a progressive risk factor. Correctly assessing the expected progression of the curvature and positively influencing it through therapy is the decisive factor of treatment. Only if a correction is successful or the expected progression is stopped, the brace treatment may be continued. If there is no progress in treatment, a stop of brace treatment has to be discussed, since a surgical intervention must not be delayed. The red flags of the indication must always be taken into account.^[1–3]^

The primary correction result in the brace is taken as a measure of the effectiveness of bracing. A correction outcome of 40%–50% is seen as the first indication of long-term improvement potential and thus becomes the strongest motivator for patient compliance. The lack of initial in-brace correction is strongly associated with brace treatment failure. However, it is self-explanatory that this result is again strongly influenced by the cause of scoliosis.^[4,5]^

The therapy goal remains unchanged the prevention of necessary scoliosis surgery. Body symmetry as a measure of the cosmetic treatment result must not be neglected. The cosmetic result is directly related to the psychological burden of scoliosis on patients.^[6]^

“Adjunctive” physiotherapy forms the basis of any conservative treatment concept, as it keeps the spine mobile and thus correctable. Whether physiotherapy supports brace treatment in its effectiveness or compensates for negative side effects of the brace treatment is in the eye of the beholder. The view that a brace “only” prolongs the physiotherapeutic measures casts many a corrective structure in a new light. What is indisputable is the interaction of both forms of treatment. Physiotherapy is essential for maintaining spinal mobility and thus correctability. The brace provides a passive but also active stimulus for scoliosis correction and has a growth-restricting effect.

A central cosmetic goal is the improvement of body symmetry, which is documented by surface representations.^[7,8]^

Due to its measurability, the compliance of the wearing time is used as a measure to evaluate the patients. However, compliance is influenced by many factors at the same time. Many different recommendations on the subject of brace-wearing duration, especially on the Internet, confuse patients, or better yet, they turn to their preferred recommendation.

Compliance, however, also concerns the practitioners: Has the medical system already failed at the initial diagnosis, or was scoliosis detected in a timely manner? Was the cause of scoliosis clarified according to the current state of knowledge? Is the therapy recommendation made convincingly and explained conclusively to the patient? Have the patient’s living conditions been taken into account and accepted in the treatment concept?

Only if the statements of the physicians, physiotherapists, orthopedic technicians, and parents are presented in unison and convincingly, can good compliance be expected from the adolescent patients. Brace-wearing time per day and the time of weaning of the brace are discussed controversially. In the literature, 12 to 16 hours of brace wear per day did not lead to a higher progression rate of AIS compared with more than 16 hours in a study group.^[9]^ The electronic recording of the brace-wearing time is the beginning of a measurable treatment process.^[10]^

## 2. Methods

A review of the literature of the last 50 years was performed from the perspective of current opinion, this with a pinch of personal experience in bracing and scoliosis surgery since 1972.

The available publications in PubMed (since 1947) are searched for the criteria scoliosis-brace and scoliosis-surgery and presented as a graph. The number of publications on braces and surgery is compared. The number of publications will be visualized in the graph of PubMed statistics. In a statement, the future scoliosis treatment is derived from the history.

The MESH terms (scoliosis, idiopathic scoliosis, adolescent idiopathic scoliosis) are presented in their number in a flow diagram and the publications on conservative therapies (brace, physiotherapy) are compared to surgical therapies (surgery).

The own patient numbers of the last 15 years are assigned according to the Cobb angle 20°–50° for a brace and >50° for surgery recommendation.

The ratio of the literature citations is compared with the number of own scoliosis patients.

Literature citations used to support history are for the past 35 years. An ethical statement is not applicable because the work deals with literature, there are no patients included.

## 3. Results

**Figure 1.**
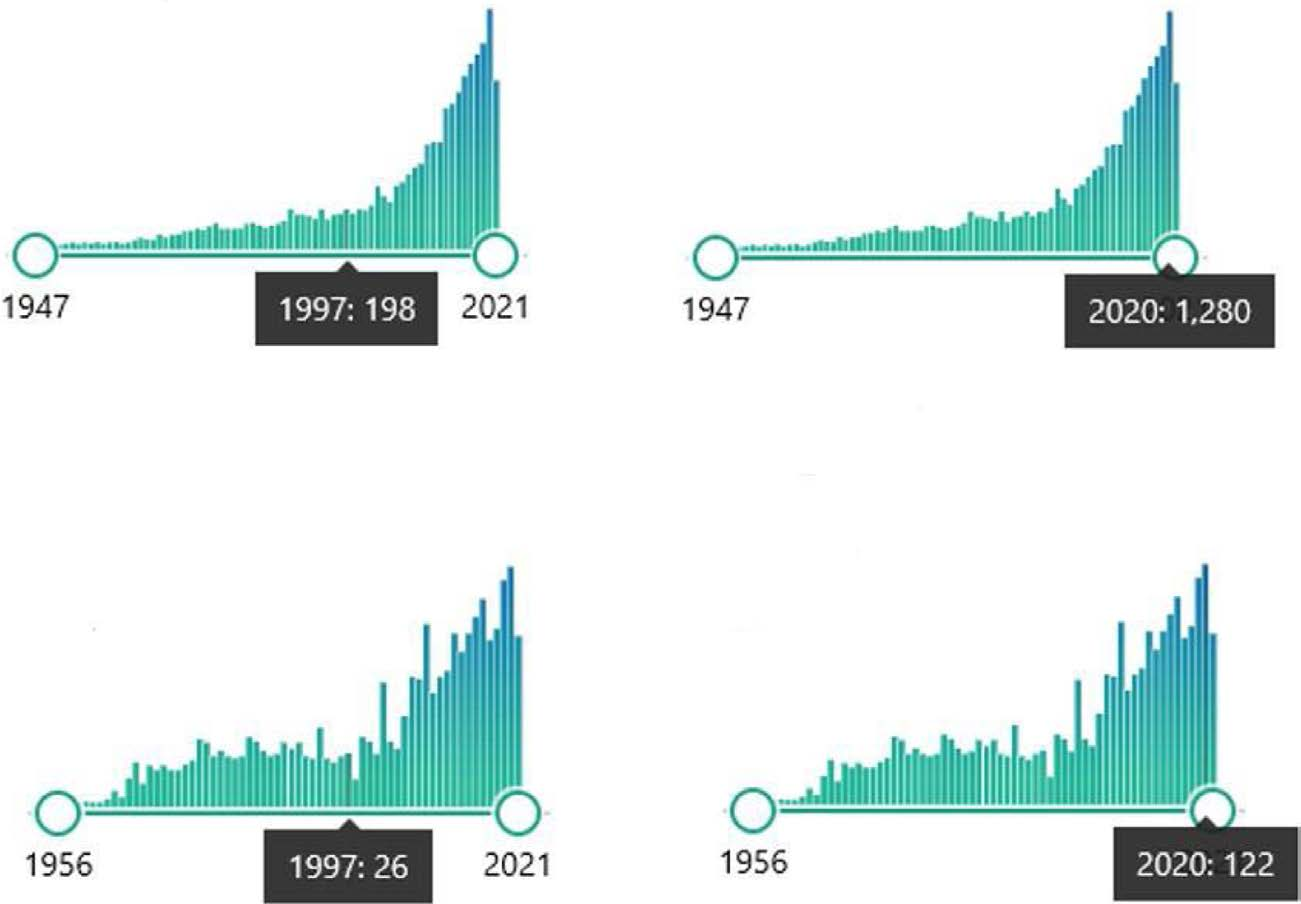
Development of citation grouped for different decades (PubMed 2021-11-15).

The graph of PubMed statistics (from 1947) shows an approximately exponential development of scientific articles for surgical measures. For publications on brace care, there is a marked depression in accepted articles on the topic of scoliosis care in the years around 1990–2000. For exemplary comparison, 198 articles were accepted on the topic of surgery in 1997, while only 26 articles were accepted on the topic of brace. In 2020, 1280 were accepted on the topic of Surgery and 122 articles on the topic of brace. Thus, the ratio of brace to surgery publications remains steady at about 1 to 10 (Fig. 1).

**Figure 2.**
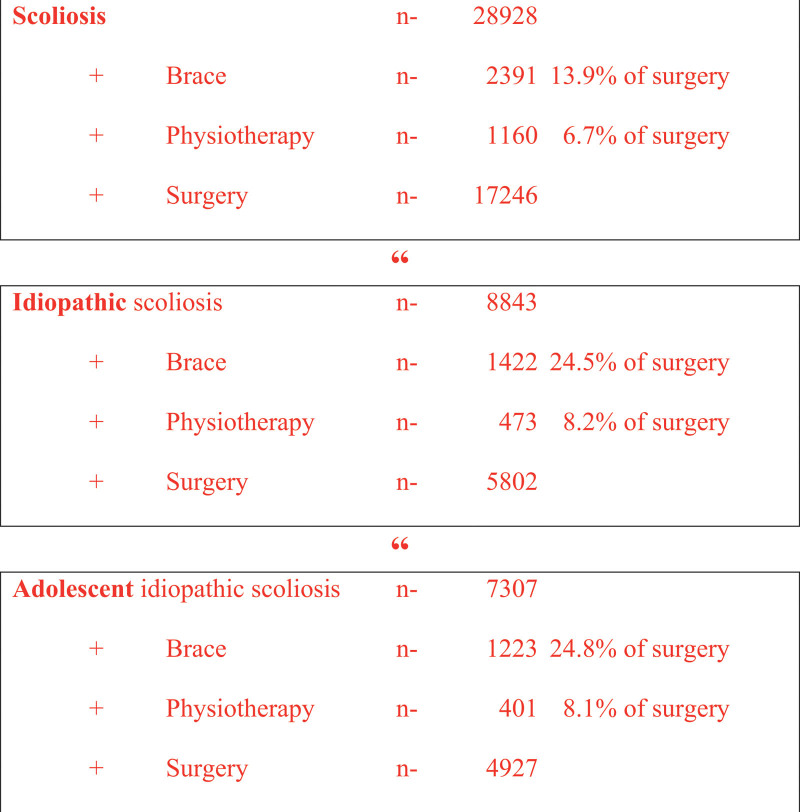
Flow diagram: MESH-Terms and number of citations (PubMed 2022-07-21).

The total number of publications shows a ratio of brace to surgery of 13.9% and physiotherapy to surgery of 6.7% for the MESH terms “scoliosis”. When “scoliosis” is supplemented with “idiopathic”, the brace to surgery ratio changes from 24.5% and physiotherapy to surgery 8.2%. Focusing on adolescent scoliosis the addition of “adolescent” changes the brace to surgery ratio from 24.8% and physiotherapy to surgery 8.1%. In the total number of publications, “adolescent idiopathic scoliosis” is treated by 25.26% (Fig. 2).

The own patient numbers of a conservatively oriented scoliosis outpatient department (1482 patients) show a ratio of brace (Cobb angle 20°–50° brace-indication of 1395 patients) to surgery (Cobb angle >50°indication to surgery of 87 patients) of 1 to 0.06 in the last 15 years. The volume distribution may be different for surgically oriented departments. However, a reversal of the ratio is not possible, since every scoliosis with a Cobb angle >50°, in its development for a long time had to be in the range of the brace recommendation of 20°–50°. The scientific focus on surgical therapy is evident from the above figures.

### 3.1. 1970–1980

At the beginning of the decade, the Milwaukee-brace was the gold standard and physiotherapy exercises were seen as an adjunctive treatment. The 23-hour wearing time was not questioned. Physiotherapy centers such as the Katharina Schroth Clinic in Germany already offer a dynamic concept. Towards the end of the decade, the Boston-brace, which starts from a symmetrical module, comes on the market. The Charleston-Bending-Brace, built as a recurving orthosis and designed for the night, shakes the dogma of 23 hours of wear.

### 3.2. 1980–1990

Jacques Cheneau’s Inspirations derotation brace emerges from this era and rapidly spreads. Many developments based on similar concepts come onto the market. Important doctors give the guidelines. It is the peak but also the beginning of the end of “eminence-based” medicine. In parallel, the possibilities of surgical scoliosis treatment develop rapidly. Even among surgeons, it is sounding names that dictate the treatment concept. The knowledge about the natural progression after growth completion becomes the generally accepted treatment goal i. e. shortened: 20°-50° Cobb angle = brace and >50° Cobb angle = surgery. This with the known gray area (overlapping indications of physiotherapy, bracing, and surgery), influenced by many concomitant diagnoses like the structure of the curve, rigidity, or family history.^[11]^

### 3.3. 1990–2000

The question of idiopathic adolescent scoliosis in its prevalence and the expected course of development is intensely debated. Thus, the proponents and opponents of conservative therapy are increasingly facing each other as referred to in Lancet 1994 by Lonstein.^[11]^

It is arguably the decade of surgery. When renowned orthopedic surgeons disparage brace treatment at the Eurospine congress with the statement: “Bracing is abuse of children,” there is little room for brace treatment. At the international congresses and in the scientific literature, the days of brace treatment seem numbered. This situation of pure advocates of only surgical procedures and supporters of conservative therapy is described very clearly by Robert B. Winter in his article “The pendulum has swung too far: Bracing for Adolescent Idiopathic Scoliosis in the 1990s. He wrote in his article: “As is typical in most of medicine, when such opposite philosophies exist, the truth lies somewhere in between. It is the proverbial pendulum in action, first swinging too far in one direction, then too far the opposite way, and finally settling in the middle.” The first signs of “evidence-based” medicine have been negated for too long (personal statement by Alf Nachemson to the author of this article). Alf Nachemson from Gothenburg is to be thanked with his study for a rethinking and for subjecting the conservative forms of treatment to a new objective evaluation. The comparison between natural progression, brace treatment, and electrostimulation led to confirmation for brace treatment but the end of electrostimulation as a treatment form.

Towards the end of the decade, EBM (evidence-based medicine) with the impact factor of the journal in which the article was published becomes the all-important quality criterion.^[12,13]^

### 3.4. 2000–2010

The answer to this development is the foundation of SOSORT (International Society on Scoliosis Orthopaedic and Rehabilitation Treatment), as a society focusing on conservative scoliosis treatment. Names such as H.R. Weiß, M. Rigo, St. Negrini, MB Grivas are mentioned as representative of this new movement. Relentless work on the subject and scientific review become the basis of a new understanding of treatment.

However, findings from the natural course of scoliosis development also provide new information for treatment goals. The creation of scientifically based treatment guidelines becomes a crucial task. In an own work on primary correction in a brace, a contribution for the evaluation of the brace design could be provided.^[14–16]^

### 3.5. 2010–2020

The 2013 work by S. Weinstein and L. Dolan becomes a watershed for the recognition of brace treatment.^[17]^ The first prospective randomized trial on the topic of brace fitting provides new momentum. The SRS (Scoliosis Research Society), as a society dominated by spine surgeons, is also rededicating itself to conservative treatment options, and collaboration between the two societies is increasingly intensified. The joint congress orientation and guideline development is a visible example of this. In the process, physiotherapy is also increasingly being subjected to scientific scrutiny. Increased attention is also being paid to the psychological burden of treatment on patients in standardized questionnaires.^[18]^ The negative impact of bracing on Quality of life is only transient as previously braced patients have superior Quality of life, compared with SRS-22r.^[19–21]^

New methods are being sought to reduce the spread in brace manufacturing. M. Rigo develops his own classification of scoliosis forms and standardization of brace construction with his database. A specific scoliosis classification that correlates with brace treatment has been proposed with an acceptable intra- and interobserver reliability.^[22]^

For the first time, C. Aubin goes one step further in the standardization of fitting quality and provides an automated correction calculation via the finite element method (FEM). Braces from the 3D printer are still at the beginning of their development.

The classification of scoliosis is thus once again the focus of interest. A study presents a new method of classifying AIS based on a fuzzy clustering algorithm using parameters describing the 3D characteristics of the deformity.^[23,24]^ The results from long-term studies are becoming increasingly decisive for the quality of treatment forms.^[25,26]^

### 3.6. Future

This is no longer a scientifically based treatise, but a contribution shaped by the professional experience of nearly 50 years. The most recent projects for quality standardization and automated calculation of the orthosis design seem to me to be unstoppable. We still see significant potential for improvement in the diagnosis of scoliosis. The availability of MRI with improved image quality, upright MRI, but also the development of video function raises high expectations for the future. For the time being, the near future is still called artificial intelligence (AI) in that the X-ray and thus the Cobb angle will be measured in an automated way, thus compensating for measurement differences between examiners. Standardization of the measurement will become the basis of quality control.

However, the importance of the Cobb angle will decrease in favor of surface symmetry as a criterion for success. This particularly affects physiotherapeutic treatments. EBM is not just a buzzword, but will also shape indication decisions. Only if the treatment is expected to be successful, the assumption of costs can be justified. This applies to both brace treatment and physiotherapy. Compliance is no longer focused solely on the patient; rather, the treatment providers are evaluated by the physician, OT, and physiotherapist according to measurable criteria.

The long-term results of conservative and surgical treatments will determine their therapeutic future.

### 3.7. Limitations

The interpretation of the literature is based on professional experience. The own scoliosis outpatient clinic has a supraregional catchment area for brace treatments. Thus, the number of brace treatments is higher than the normal distribution would suggest. However, this is not expected to change the overall picture in the ratio of brace treatment to surgical treatment. Any scoliosis with a Cobb angle >50° was in its development for a long time in the range of the brace recommendation of 20°–50°.

## 4. Discussion

The question remains: Why is little attention paid to conservative treatment modalities before achieving surgical criteria?

After all, every idiopathic scoliosis requiring surgery has gone through all stages of development from the diagnosis of scoliosis (Cobb angle 10°), the recommendation for physiotherapy and brace recommendation to the recommendation for surgery (Cobb angle >50°).

The causes of delayed initial diagnosis (screening program) are currently inconclusive in the literature.

The effectiveness of conservative scoliosis treatment is assured. However, the chances of success in individual cases can only be predicted to a limited extent. There is no classification for promising curvatures that can be treated conservatively.

The word “idiopathic” is used very generally, i.e. a clarification algorithm for the cause of scoliosis is required.

It is to be expected that the acceptance of conservative treatments would be much better if only promising forms of curvature were also submitted to treatment. In patients with very unfavorable curvatures with no expected success of conservative treatment, the burdensome conservative treatment should not be initiated.

## 5. Conclusion

It is like a small miracle that conservative scoliosis treatment has managed to free itself from the rejection of the 90s. The consistent scientific evidence is paying off. This scientific consistency must also be demanded for other forms of orthopedic treatment. Only in this way will it be possible to maintain the status of orthosis. In this sense, the brace treatment of the last 50 years is a success story with positive prospects. The central message from the history of the last 50 years is: “The scientific review of treatment results is essential for the further acceptance of brace treatment.“

Robert B Winter was right when he wrote in 1994: “It is the proverbial pendulum in action, first swinging too far in one direction, then too far the opposite way, and finally settling in the middle.”^[12]^

## Author contributions

FL and KT wrote and conceptualized the work.
